# The Neurobiological Mechanism of Chemical Aversion (Emetic) Therapy for Alcohol Use Disorder: An fMRI Study

**DOI:** 10.3389/fnbeh.2017.00182

**Published:** 2017-09-28

**Authors:** Ralph L. Elkins, Todd L. Richards, Robert Nielsen, Richard Repass, Henriettae Stahlbrandt, Hunter G. Hoffman

**Affiliations:** ^1^Department of Medical Research, Schick Shadel Hospital, Seattle, WA, United States; ^2^Department of Radiology, Integrated Brain Imaging Center, University of Washington, Seattle, WA, United States; ^3^Human Photonics Lab, Mechanical Engineering, University of Washington, Seattle, WA, United States

**Keywords:** alcohol, opioid, craving, fMRI, aversive conditioning, alcohol treatment, addiction, craving-related brain activity

## Abstract

A recent NIH epidemiology study found the lifetime prevalence of alcohol use disorder in the United States to be 29%. Alcohol drinking behavior is strongly “learned” via pleasure center activation/reinforcement. Alcohol craving is a powerful desire to drink alcoholic beverages. Craving was added as one of the defining criteria for alcohol use disorder in DSM5, and craving reduction is becoming an increasingly important treatment goal. In the current study, patients with alcohol use disorder received 10 days of inpatient multi-modal treatments at Schick Shadel Hospital (SSH) of Seattle. The treatments included five chemical aversion conditioning sessions that associated alcohol cues (and alcohol) with nausea and emesis. All patients met DSM4 criteria for alcohol use disorder, were heavy drinkers, and reported craving alcohol pre-treatment. Craving reduction was one of the primary treatment goals. This is the first fMRI study to measure the effects of chemical aversion therapy on alcohol craving-related brain activity. Patients were recruited as subjects for the University of Washington (UW) brain scan study following SSH admission but before treatment onset. Prior to treatment, patients reported craving/desire for alcohol. After treatment (after four SSH chemical aversion treatments, again after five SSH chemical treatments, 30 and 90-days post-discharge), these same patients reported avoidance/aversion to alcohol. Most of the participants (69%) reported being still sober 12 months post-treatment. Consistent with a craving reduction mechanism of how chemical aversion therapy facilitates sobriety, results of the UW fMRI brain scans showed significant pre- to post-treatment reductions in craving-related brain activity in the occipital cortex. Additional fMRI brain scan studies are needed to further explore the neurobiological mechanism of chemical aversion therapy treatment for alcohol use disorder, and other substance use disorders for which chemical aversion therapy is used (e.g., opioid dependence and cocaine dependence). Substance use disorders are estimated to affect well over one billion people worldwide.

## Introduction

Alcohol-use disorders, which include both alcohol abuse and dependence, make up one of the most prevalent categories of substance use disorders. According to a recent NIH epidemiological study, the lifetime prevalence of alcohol use disorder in adults 18 and older in the United States is 29% (Grant et al., 2015). Alcohol dependence is “a maladaptive pattern of drinking leading to clinically significant impairment, as manifested by a compulsion to drink, a lack of control over urges to drink (craving), a lack of control over the amount of alcohol consumed, and continued drinking despite realization of the associated problems,” (The Diagnostic and Statistical Manual of Mental Disorders-Fourth Edition, DSM-4; American Psychiatric Association, 2000). According to the DSM-5 (American Psychiatric Association, 2013), activation of the brain's reward (pleasure) system is a major source of problems for alcohol users. People with alcohol use disorder experience a euphoric high feeling after drinking alcohol. Human brains have evolved to reward life-sustaining activities to encourage organisms to repeat those behaviors. When the reward center of the brain activates, the organism is more likely to remember and repeat the behavior they are currently performing at the time, without even thinking about it (Ostafin et al., 2008). Unfortunately, when the behavior is drinking alcohol, the alcohol drinking behavior is strongly “learned” via pleasure center activation/reinforcement. The pleasure of drinking can be so rewarding that the people with alcohol use disorder focus on behaviors that repeat this experience (e.g., drinking) at the expense of other more important behaviors.

Chronic alcohol users develop stimulus-response conditioned habits. They learn to associate various people, situations, and places with drinking alcohol/euphoria. Even after treatment, when people encounter these stimulus cues in their daily life, alcohol cues can cause craving. Simply looking at pictures of people drinking alcohol or seeing alcoholic beverages or even fantasizing/visualizing themselves drinking their favorite alcoholic beverage may cause people with alcohol use disorder to feel mild pleasure effects typically associated with alcohol use, almost as if they have taken a small drink. Many people follow that inclination and start drinking alcohol again (as reviewed in Tapert et al., 2004). As mentioned, the person often resumes drinking without even thinking much about it, as a stimulus-response conditioned reflex (Ostafin et al., 2008; Kreusch et al., 2017). Patients who quit drinking alcohol (detox) without treatment have a high risk of relapse (e.g., 80% relapse rate, Heinz et al., 2009). Research studies show correlations between craving (urge to take a drink of alcohol) and severity of alcohol dependence (Yoon et al., 2006). Craving in response to viewing alcohol related images has been used to predict probability of relapse (Heinz et al., 2009; Papachristou et al., 2014). The higher the patient's subjective ratings of craving pre-treatment, the more severe the dependence, and the greater the likelihood the patient will resume drinking alcohol after treatment. Treatments for alcohol use disorder that target craving may be especially effective for patients who crave alcohol.

Craving reduction is becoming an increasingly important goal of treatments for alcohol use disorder (Casey et al., 2012; Field and Jones, 2017; Roberts et al., 2017). According to Casey et al. (2012, p. 76) “The development of pharmacologic anti-craving interventions….often used as adjuncts to verbal therapies, have also been shown to be effective in reducing craving in patients, resulting in improved treatment outcome.” Some treatments that target craving have been shown to significantly reduce heavy drinking post-treatment (Witkiewitz et al., 2011; Casey et al., 2012; Bowen et al., 2014; Cabrera et al., 2016). As an example of the growing priority given to craving, the DSM-5 (American Psychiatric Association, 2013) added craving as one of the diagnostic criteria of alcohol use disorder.

The current study explores the use of emetic counter conditioning that targets alcohol craving. Fortunately, in addition to learning to repeat behaviors associated with pleasure center activation (pathological craving/desire for alcohol), the brain also has a mechanism for enhanced learning to avoid behaviors associated with nausea and vomiting. Humans and other animals learn to avoid eating poisonous foods via a taste aversion conditioning mechanism (Elkins, 1980). In the current study, patients with alcohol use disorder received a treatment that included alcohol/taste aversion conditioning (Elkins, 1991a,b; Smith and Frawley, 1993; Smith et al., 1997; Howard, 2001; Frawley and Howard, 2013; Frawley et al., 2017). This chemical (emetic) aversion therapy specifically targets unconscious/habit memory associations/alcohol craving. Craving reduction is one of the primary treatment goals.

Several brain scan studies have shown alcohol craving-related brain activity in the “reward circuitry” of the brain (see Courtney et al., 2016 for review). For example, using a gustatory alcohol cue reactivity paradigm, Courtney et al. (2015) reported alcohol-cue related brain activity in the following five regions on the brain: the hippocampus, amygdala, inferior frontal gyrus, temporal cortex, and occipital cortex. A meta-analyses by Hanlon et al. (2014) showed that the occipital cortex was activated in 86% of the alcohol research studies. In the current study, we predicted that after the first 8 days of a 10 day in-house treatment for alcohol use disorder focusing on taste aversion counter conditioning, patients who craved alcohol pre-treatment would report reductions in how much they craved alcohol after treatment. In addition, fMRI brain scans were used to quantify for the first time whether chemical alcohol/taste aversion conditioning reduces alcohol cue related brain activity, toward a better understanding of the neurobiological mechanism of this treatment. Using whole brain analysis, we predicted reductions in self-generated alcohol cue-related brain activity after treatment.

## Methods

This study had IRB approval from the University of Washington in Seattle. Patients signed written informed consent forms prior to the study. All patients met DSM-4 (American Psychiatric Association, 2000) criteria for alcohol use disorder, and reported craving alcohol pre-treatment. All clinical alcohol use disorder treatments were conducted at Schick Shadel by Schick Shadel staff. The University of Washington, Seattle (UW), was not involved in treating the patients. Patients already diagnosed and admitted to Schick Shadel Hospital for treatment of alcohol use disorder were identified as potential study subjects during their initial medical assessments by SSH physician coauthors (K.D. and R.R). These patients were offered the opportunity to participate in a craving and fMRI research study to be conducted at the University of Washington Seattle. The prospective participants were informed that the study was designed to measure craving and craving-related brain activity before beginning treatment, and after 8 days of the 10 day in house treatment for Alcohol Use Disorder at Schick Shadel Hospital in Seattle. All fMRI scans were conducted at the UW fMRI Research Laboratory center at Diagnostic Imaging Sciences Center/Integrated Brain Imaging Center (DISC/IBIC).

### Study participants

The 13 participants were aged 29–55 years of age (Mean = 45 years old, *SD* = 6.83, 23% female, 77% male), with alcohol use disorder, who had sought treatment and had already entered themselves into the 10 day Schick Shadel treatment for Alcohol Use Disorder, and had had not used alcohol during the 48 h before their pre-treatment fMRI brain scan. According to patients self-reports, the mean “duration of alcohol disorder” of the patients scans was 18.7 years (*SD* = 8.11, range 10–34 years). Sixteen subjects (13 males and 3 females) initially participated in the first scan after passing the pre-test screening. Three subjects (3 males) did not receive the post-treatment fMRI scan.

Patients were recruited to participate in the current fMRI brain scan study at the University of Washington. The inclusion criteria were as follows: SSH admissions who had received a DSM-4 (American Psychiatric Association, 2000) diagnosis for alcohol use disorder, and had reported craving alcohol. Consenting patients were further screened for eligibility via the MRI safety questionnaire, and a claustrophobia screening questionnaire.

### Exclusion criteria were as follows

A history of psychiatric disorder, multi-substance use disorder, a history of neurological illness, previous head trauma resulting in loss of consciousness greater than 2 min, serious medical problems (including contraindications to chemical counter conditioning treatments), learning disability, current use of medications that could affect the central nervous system, significant maternal drinking during pregnancy, family history of bipolar or psychotic disorders, inadequate English skills, left handedness, contraindications to MRI scans (e.g., irremovable metal on the body), claustrophobia, and no strong craving to alcohol pre-treatment.

**Figure 1:**
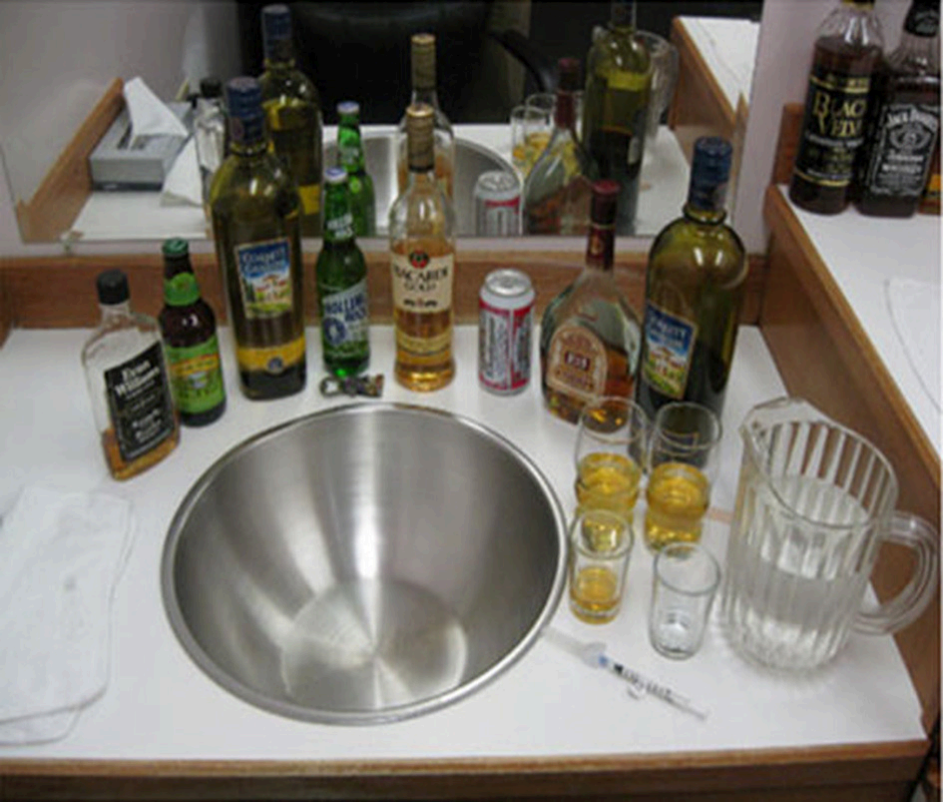
A typical setup of an alcohol treatment room.

The treatment itself was “standard of care, treatment as usual” at Schick Shadel, including individual and group counseling plus educational lectures similar to those used in many inpatient treatment programs. However, Schick Shadel also features five unique anti-craving counter conditioning treatments designed to change alcohol cravings into alcohol aversions/revulsions. When the patient entered the treatment room, he/she sat before the emetic basin (bathroom sink, see Figure 1) and saw a display of the preferred alcoholic beverage containers.

Ipecac is available as an FDA approved drug used to induce vomiting. The emetic drug is obtained from the dried roots of a plant named Carapichea ipecacuanha.

Emetine is the primary emetic compound of Ipecac. Although a number of emetic drugs can be used for aversion conditioning (e.g., disulfiram), as summarized by Howard et al. (1991), p. 135, “emetine is the agent that most reliably produces nausea and emesis; (2) emetine is effective, vis-a-vis production of conditioned aversion and is relatively innocuous (Loomis et al., 1986).”

During the taste aversion conditioning session, patients repeatedly tasted and swallowed alcoholic beverages that were promptly expelled via ipecac induced nausea and emesis (for details see Elkins, 1980; Frawley et al., 2017). The chemical counter conditioning treatments were continued on an every-other-day basis with adjustments to the nausea inducing drug dose as needed, until five treatments had been completed. The patient was discharged after the 10-day treatment, and asked to return to the hospital for overnight stays including booster chemical aversion treatments at 30 and 90 days post-discharge. The hospital continued routine follow up contacts for a year post-discharge (see Elkins, 1980; Frawley et al., 2017). Sobriety was assessed 12 months post-discharge.

### fMRI scans

Subjects received their pre-treatment fMRI brain scan at the University of Washington during self-generated alcohol stimulus cues before any treatment onsent, and they received one post-treatment fMRI brain scan after 8 days (after four of the five counter conditioning treatments) of the standard 10 day Schick Shadel treatment for alcohol use disorder. Alcohol craving was assessed during the 30 min that preceded each of the two fMRI sessions. We elected to complete the second and final UW session following four instead of five SSH chemical aversion treatments to insure that an additional SSH chemical aversion treatment would be available in the unexpected event that our UW procedures induced “longer than intended” alcohol cravings in any of the patient subjects. Patients answered single item questions (e.g., Bujarski et al., 2015) about their current level of “wanting, liking, and craving alcohol” (three single item questions). They received the following instructions. “Please indicate how you feel about alcohol right now, by making a slash through the appropriate number below. You can select a whole number or make a mark between numbers as appropriate (i.e., your answer does not have to be a whole number).” The reliability and validity of the Graphic Rating Scales have been shown in a variety of adult patient populations (Jensen and Karoly, 2001).

Before each fMRI scan, patients also answered a single Likert based Desire/Aversion Scale, “how do you feel about alcohol right now” (0 = strong aversion, 10 = strong craving/desire). Before receiving any treatment, and after 8 days of treatment at Schick Shadel hospital, patients were also asked to answer 12 brief rating questions on a scale from 1 to 7 about alcohol craving, using the Alcohol Craving Questionnaire, Short Form Revised (ACQ-SF-R), Singleton et al. (2000). For example, “if I had some alcohol, I would probably drink it,” 1 = strongly disagree, 7 = strongly agree. The ACQ-SF-R contains 12 items strongly correlated with the total ACQ score. The ACQ-SF-R has moderate to high reliability and is sensitive to change (Singleton et al., 2000; see also Raabe et al., 2005). Three of the questions were reverse ordered, as appropriate, before summing to obtain a total score.

### fMRI procedures and assessment

All fMRI scans were acquired using a standard protocol on a research dedicated 3T Philips Achieva (version 3.2.2) using a blood oxygenation level-dependent (BOLD) EPI pulse sequence with the following parameters: repetition time (TR) = 1,988 ms, echo time (TE) = 35 ms, flip angle = 90, FOV = 224 × 224 × 132.6 mm, voxel size = 2.8 × 2.8 × 3.6 mm^3^, number of slices = 37, EPI factor = 41, no slice gap; number of dynamics/volumes = 234. The participant first underwent a series of structural scans of ~20 min total in length. Shortly after the structural MRI (without removing the participant from the scanner), the fMRI data were acquired using an on-off brain scan paradigm (adapted from Hoffman et al., 2004, 2007).

Each subject's drink of choice and a preferred drinking setting were determined by R.L.E. during an interview at SSH prior to the patients first session at the UW. Also identified were patient-specific pleasant scenes that had nothing to do with alcohol ingestion. Each patient came up with their own “non-alcohol” setting. Typically such scenes included sitting on a beach, enjoying a familiar mountain view, reading a favorite book or enjoying dinner with family members. This information was communicated to T.R. and H.G.H for use during fMRI assessments of patients' brain activity during imagined/self-generated drinking experiences.

**Figure 2:**
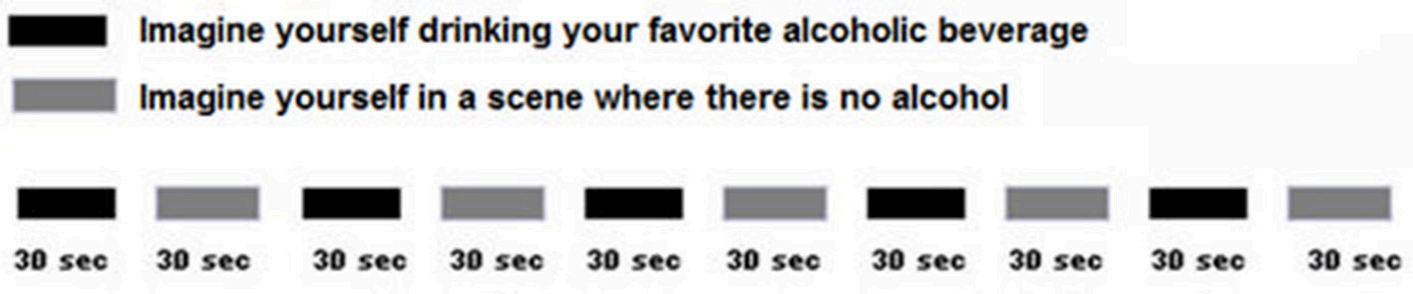
During fMRI, patients self-generated images of themselves enjoying their favorite drink, and then self-generated images of themselves in a scene with no alcohol. This was designed to measure alcohol craving related brain activity. After pre-treatment craving ratings and their pre-treatment fMRI scans at the University of Washington, Seattle, patients returned to SSH where they participated in routine treatments for alcohol use disorder.

During the 10 min fMRI brain scan, patients alternated between self-generating images of drinking alcohol, and self-generating neutral images of themselves with no alcohol, in response to verbal suggestions (see Figure 2). Self-generated/imagined substance exposure scenes (in our case drink of choice scenes) were alternated with self-generated images of relaxing scenes that involved no substance use. This alternation of drink of choice and control scenes allowed fMRI measurement of alcohol craving-related brain activity.

As an example, the following are verbal suggestions that would be appropriate for a patient whose drink of choice is Teachers Scotch (Teachers is a brand). Each suggestion is followed by temporal duration information. “*I want you to imagine that you see a bottle of Teachers Scotch before you and that you have some scotch in your mouth that you taste and perhaps smell.”* The suggestion took ~15 s. and the scene lasted 45 s. from onset, thereby providing about 30 s. for the patient's imagination participation. The following sample control suggestion features a beach scene. Each actual control suggestion was subject specific and based on a preferred scene from the patient's memory. The only requirements were that the imagined setting should be pleasant and should have nothing to do with alcohol ingestion. “*I want you to imagine that you are sitting on your favorite beach and enjoying the white sand, sun and waves.”* The next suggestion reinitiated the sequence with a repeat of the second Teachers Scotch scene. During the ~10 min fMRI stimulus set, instructions guided each subject though five cycles of alternating imagined/self-generated personal drinks-of-choice or imagined/self-generated neutral control (no alcohol) scenes as previously instructed to participants.

## Results

Using paired *t*-tests, patients reported large and statistically significant pre- to post-treatment reductions for wanting, liking, and craving alcohol (see Table 1).

**Table 1:** Patients' ratings/responses on a subjective graphic rating scale questionnaire (three questions), before and after treatment.

**Mean pre-treatment**		**Mean post-treatment**	
Want alcohol	4.46	0.00	*t*_(12)_ = 4.28, *p* < 0.005, *SD* = 3.76
Alcohol appealing	4.92	0.08	*t*_(12)_ = 4.85, *p* < 0.005, *SD* = 3.83
Crave alcohol	3.85	0.00	*t*_(12)_ = 3.84, *p* < 0.005, *SD* = 3.69
ACQ^a^	33.09	14.91	*t*_(10)_ = 3.54, *p* < 0.01, *SD* = 17.06

a *The mean total score on the Alcohol Craving Scale-SF-R*.

**Figure 3:**
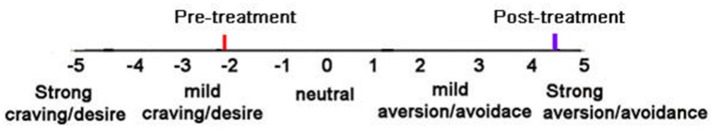
Patients subjective ratings on the “craving/desire vs. aversion/avoidance” rating scale before treatment and after 4 aversion therapy sessions (post-treatment).

Before their first chemical aversion treatment, patients reported a moderate desire/craving for alcohol, (see Figure 3). After four chemical aversion conditioning sessions at SSH, patients reported a strong aversion to alcohol [mean before = −2.17 before vs. +4.44 after four chemical aversion treatments, *t*_(12)_ = 9.36, *p* < 0.001, *SD* = 2.50]. The patients further received booster chemical aversion conditioning sessions at 30 and at 90 days post-discharge, during overnight hospital visits. Patients still reported strong aversion/avoidance to alcohol immediately before their booster session at 30 days and immediately before their booster session 90 days post-discharge. Paired *t*-tests compared ratings “before treatment” vs. craving/aversion at 30 days post-treatment, [mean = −2.39 before vs. +4.25 after, *t*_(12)_ = 10.25, *p* < 0.001, *SD* = 2.37]. Paired *t*-tests compared ratings “before their first chemical aversion treatment” (mean = −2.46 before vs. +4.27 after 90 days), *t*_(10)_ = 7.51, *p* < 0.001, *SD* = 2.97.

### Sobriety

According to Schick Shadel's standard follow-up assessments, when contacted at 1 year post-treatment, 69% of the 13 patients in our fMRI study above reported still being sober/had not relapsed at 1 year post-treatment followup, 15.4% relapsed, and 15.4% could not be reached at 1 year followup, and are unknown (and are presumed non-sober).

In summary, patients reported mild desire/craving for alcohol before treatment. After the first four chemical aversion treatments, the patients now reported a strong aversion/repulsion to alcohol, and this strong aversion to alcohol was still evident 30 and 90 days post-treatment. Furthermore, 69% of the participants reported being abstinent 12 months post-treatment.

### Results from the fMRI brain scans

**Figure 4:**
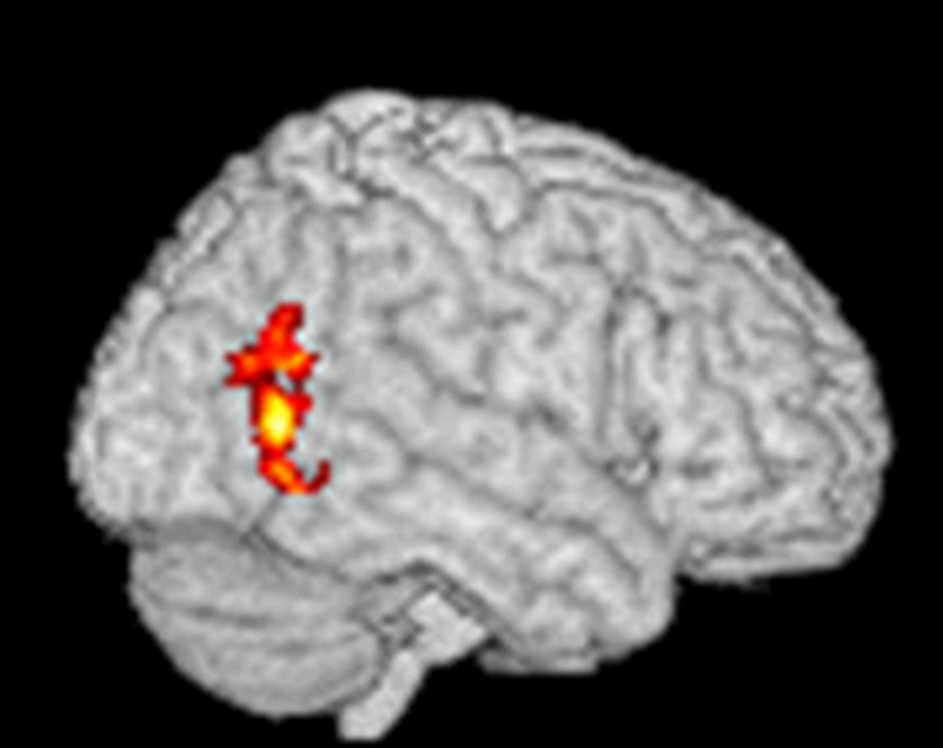
Statistical fMRI image showing the areas of brain where there was a significant paired decrease from pre-treatment to post-treatment using verbally cued imagined alcoholic beverage craving stimuli to elicit craving-related brain activation before and after aversive conditioning. The red areas show where there was a significant change in fMRI at a corrected *p*-value < 0.05 and the yellow areas show where there was a significant change in fMRI at a corrected *p*-value < 0.03.

Using an fMRI paradigm designed to measure craving-related brain activity, patients received verbally guided self-generated visualizations of drinking alcohol vs. visualizing non-alcohol scenes during fMRI brain scans before and after four chemical aversion treatments for alcohol use disorder. Compared to pre-treatment, at post-treatment, patients reported significant post-treatment reductions in how much they craved alcohol, and, consistent with our prediction that chemical aversion therapy would reduce craving, their post-treatment fMRI brain scan showed significant reductions in alcohol cue-related brain activity in the occipital cortex (as shown in Figure 4). FMRI statistical images were calculated using FSL's randomize software using the threshold-free cluster enhancement option using a design matrix as shown in this website https://fsl.fmrib.ox.ac.uk/fsl/fslwiki/FEAT/UserGuide#PairedTwo-Group_Difference_.28Two-Sample_Paired_T-Test.29 for paired data. This software does compensate for multiple comparisons and using subject-based permutations to develop the proper statistical distribution to calculate p values. The pre- to post-treatment reduction in alcohol craving related brain activity in the occipital cortex was statistically significant at a corrected *p*-value < 0.05. Cluster Index = 1, Voxels = 439, *p* = 0.043, −log10(*P*) = 1.36, Z-MAX = 3.84, Z-Max X (mm) = 40, Z-Max Y (mm) = −62, Z-MAX Z (mm) = 6, COPE-MAX = 13.2, COPE-MEAN = 7.63. Note that the X, Y, Z coordinates are in MNI space.

## Discussion

The present study measured how much subjects craved alcohol (subjective ratings of craving) before and after completing 8 days (i.e., four aversion treatments) of a 10 day inpatient alcohol use disorder chemical aversion treatment program. Before treatment, patients reported having mild craving/desire for alcohol. After completing 8 days of treatment, these same patients reported avoidance/aversion to alcohol. During follow up measures 30 and 90 days post-discharge, patients still reported strong aversion to alcohol. Furthermore, according to Schick Shadel's standard follow-up assessments, when contacted at 1 year post-treatment, 69% of the patients in our fMRI study above reported still being sober at 1 year post-treatment followup. Although some relapses occur without a significant explicit desire/craving, previous studies have shown that patients who crave alcohol are at increased risk of relapse, after treatment. For treatments that don't specifically target craving, craving for alcohol is a predictor of poor outcome (Yoon et al., 2006). In contrast, in the current study, craving for alcohol was a prerequisite to be eligible to participate in this study. All of the participants in the current study reported craving for alcohol, pre-treatment. After treatment, these same individuals reported avoidance/aversion to alcohol. Chemical aversion therapy is a technique that targets craving reduction as one of the primary outcome goals. This treatment may be especially effective for treating patients who crave alcohol.

In addition to chemical aversion conditioning, during therapy session and post-treatment follow up visits, patients learn relapse prevention strategies to help them cope with craving/desire and other relapse risks. For example, they learn to identify and avoid/get out of high risk situations (e.g., Larimer et al., 1999). With some types of treatments, patients who crave alcohol may be trained to use that craving as a conscious warning that they are entering a situation that puts them at increased risk of relapse. However, the treatment used in the current study appears to work by reducing craving, and even causing the patients to become aversive/avoidant of alcohol when exposed to alcohol cues. To facilitate relapse prevention, as standard of care at Schick Shadel Hospital, patients returned to the hospital to receive booster chemical aversion conditioning sessions and in house CBT therapy sessions at 30 and at 90 days post-discharge, during overnight hospital visits. Patients still reported strong aversion/avoidance to alcohol immediately before their booster session at 30 days and immediately before their booster session 90 days post-discharge.

This is the first published study to measure the effects of chemical aversion therapy on craving-related brain activity. Courtney et al. (2015) reported alcohol cue related brain activity in the following five regions of the brain: the hippocampus, amygdala, inferior frontal gyrus, temporal cortex, and occipital cortex. A meta-analyses by Hanlon et al. (2014) showed that the occipital cortex was activated in 86% of the alcohol research studies in the literature. In the current study, we predicted that after four chemical aversion therapy sessions during the first 8 days of Schick Shadel Hospital's in-house treatment for alcohol use disorder focusing on taste aversion counter conditioning, patients who craved alcohol pre-treatment would report reductions in how much they craved alcohol after treatment, and their fMRI brain scans would show reductions in alcohol cue-related brain activity after treatment. Consistent with a craving reduction mechanism of how chemical aversion therapy facilitates sobriety, results of our fMRI brain scans showed statistically significant reductions in craving-related brain activity in the occipital cortex.

There are limitations that should be kept in mind when interpreting these results. As is often the case in brain scan studies, the size of the sample of the current study was relatively small, which may limit generalizability. Alcohol craving studies typically show craving related brain activity in the “reward center” of the brain, which includes several brain regions (e.g., Courtney et al., 2016). In contrast, the current study showed reductions in craving related brain activity in only one region: the occipital cortex. Although the occipital cortex is often associated with craving, the occipital cortex is not usually considered part of the “reward circuitry”. The role played by the occipital cortex in craving and alcohol use disorder is not well-understood. Although patients had not started the chemical aversion therapy sessions before their “pre-treatment” fMRI scan, patients had already undergone 48 h of sobriety before their first fMRI scan. In retrospect, this sobriety may have reduced craving-related brain activity pre-treatment (e.g., De Sousa Fernandes Perna et al., 2017). If so, the current results underestimate the magnitude of how much the full in house therapy reduced craving and reduced craving related brain activity. To obtain stronger (more accurate) craving related brain activity pre-treatment, future studies may consider having patients undergo their first fMRI scan before beginning sobriety. Future studies should also consider using larger samples sizes, and stronger stimulus cues during the fMRI brain scans (e.g., tiny tastes of the patient's favorite alcoholic beverage vs. non-alcoholic beverage squirted into their mouth on/off during fMRI brain scans, Courtney et al., 2015). Another limitation is the current study's reliance on patients to accurately self-report their post-treatment alcohol use behavior (if any) at 1 year follow up. According to a review of fMRI studies on substance use disorders (Courtney et al., 2016), alcohol relapse is frequently measured using only patient self-report, and there is evidence for the validity of self-report measures of alcohol use disorder relapse (Simons et al., 2015). However, in future studies, it would be valuable to more rigorously establish the long term treatment efficacy of chemical aversion therapy (e.g., verification of abstinence via friends and family reports, breathalyzers blood tests, and urine tests). According to Whitford et al. (2009), collateral informants can help support the validity of self-report of abstinence vs. relapse.

Despite these limitations, the current study makes an important contribution to the literature. Results provide strong evidence that chemical aversion conditioning greatly reduces subjective craving ratings, turning alcohol craving/desire into alcohol aversion/avoidance, in a sample of patients diagnosed with alcohol use disorder who were heavy chronic alcohol users, and reported craving alcohol pre-treatment. Aversion conditioning involves deliberately associating alcohol cues (and alcohol) with nausea and emesis. As reviewed in the introduction, craving reduction is becoming an increasingly important goal of treatments for alcohol use disorder (Casey et al., 2012; Field and Jones, 2017). As an example of the growing priority given to craving, the DSM-5 (American Psychiatric Association, 2013) added craving as one of the diagnostic criteria of alcohol use disorder. As mentioned earlier, previous studies have shown correlations between craving (urge to take a drink of alcohol) and severity of alcohol dependence (Yoon et al., 2006). For example, craving in response to viewing alcohol stimulus cues has previously been used to predict probability of relapse (Heinz et al., 2009; Papachristou et al., 2014). Unless craving reduction is targeted by the treatment, the higher the patient's subjective ratings of craving pre-treatment, the more severe the dependence, and the greater the likelihood the patient will resume drinking alcohol after treatment. Chemical aversion treatment specifically targets craving. The current study shows evidence that after therapy involving chemical aversion, exposing patients to alcohol cues no longer elicited craving, but instead elicited aversion/avoidance. Further research on this topic is warranted, and may lead to a better understanding of the neurobiological mechanism of chemical aversion conditioning for alcohol use disorder, and other substance use disorders for which chemical aversion therapy is being used, such as opioid dependence and cocaine dependence (Frawley et al., 2017). Since substance abuse disorders are now estimated to affect well over one billion people worldwide, more research is needed.

## Ethics statement

This study was carried out in accordance with the recommendations of University of Washington Human Subjects Division, Committee B, with written informed consent from all subjects. All subjects gave written informed consent in accordance with the Declaration of Helsinki. The protocol was approved by the University of Washington Human Subjects Division, Committee B.

## Author contributions

All authors listed, have made substantial, direct, and intellectual contribution to the work, and approved it for publication.

### Conflict of interest statement

The authors declare that the research was conducted in the absence of any commercial or financial relationships that could be construed as a potential conflict of interest.
